# INDEX-db: The Indian Exome Reference Database (Phase I)

**DOI:** 10.1089/cmb.2018.0199

**Published:** 2019-03-06

**Authors:** Husayn Ahmed P, Vidhya V, Ravi Prabhakar More, Biju Viswanath, Sanjeev Jain, Mahendra S. Rao, Odity Mukherjee

**Affiliations:** ^1^ Accelerator Program for Discovery in Brain Disorders Using Stem Cells (ADBS), National Centre for Biological Sciences, Tata Institute of Fundamental Research (NCBS-TIFR), Bengaluru, India.; ^2^ Institute of Bioinformatics and Applied Biotechnology (IBAB), Bengaluru, India.; ^3^ Accelerator Program for Discovery in Brain Disorders Using Stem Cells (ADBS), Centre for Brain Development and Repair (CBDR), Institute for Stem Cell Biology and Regenerative Medicine (InStem), Bengaluru, India.; ^4^ Department of Psychiatry, National Institute of Mental Health and Neuro Sciences (NIMHANS), Bengaluru, India.

**Keywords:** genetic variations catalogue, Indian population, population-specific database, whole exome sequencing

## Abstract

**Deep sequencing-based genetic mapping has greatly enhanced the ability to catalog variants with plausible disease association. Confirming how these identified variants contribute to specific disease conditions, across human populations, poses the next challenge. Differential selection pressure may impact the frequency of genetic variations, and thus detection of association with disease conditions, across populations. To understand genotype to phenotype correlations, it thus becomes important to first understand the spectrum of genetic variation within a population by creating a reference map. In this study, we report the development of phase I of a new database of genetic variations called INDian EXome database (INDEX-db), from the Indian population, with an aim to establish a centralized database of integrated information. This could be useful for researchers involved in studying disease mechanisms at clinical, genetic, and cellular levels.**

## 1. Introduction

The human population has increased significantly in numbers across all geographical regions in the recent past, resulting in population-specific genetic architecture. Such rapid population growth has a significant impact on the occurrence and frequency of genetic variations, especially rare variants that may lie on conserved protein encoding sites, which may have a likely role in disease biology (Keinan and Clark, 2012). Next-generation sequencing (NGS) strategies have greatly improved the ability to identify genetic variants of varying frequencies. Recent studies to identify genetic variants associated with common noncommunicable diseases suggest that these syndromes have high heritability and that the risk arises from a polygenic contribution caused by a combination of rare deleterious and common polymorphic modifier variants. NGS-based evaluation of disease association thus becomes a useful way to identify the genetic signature of a disease. A critical component of this analysis is the assignment of pathogenic relevance to the identified variants done primarily by defining the frequency in affected individuals compared with control healthy samples.

In this context, several genetic variation databases have been established, incorporating different strategies and technological improvements [e.g., haplotype mapping—HapMap project (The International HapMap Consortium, 2005); whole-genome sequencing—1000 Genomes project (The 1000 Genomes Project Consortium, 2015); and whole-exome sequencing (WES)—Exome Aggregation Consortium (Lek et al., 2016)]. While information from these databases improved our understanding of the complexities of the genetic architecture, it is also reported that a significant proportion of the genetic variations identified are population specific. We thus need a detailed evaluation in diverse populations to better understand the epidemiology and semiology of human diseases and their relationship with genes that confer susceptibility (Craddock and Owen, 2010; Bamshad et al., 2011; Hindorff et al., 2011; Higasa et al., 2016).

The Indian subcontinent has already witnessed a steep increase in the number of individuals needing care for common adult-onset disorders due to improved health care and life expectancy (a threefold increase in 60 years). Identification of a disease-specific genetic signature is a critical first step in identifying (1) disease-associated genetic variations, (2) molecular subtyping of complex human phenotypes, and (3) at-risk individuals with improved efficiency. A comprehensive reference variation map, established from clinically normal individuals who are representative of this population, will be of great benefit. There have been several reports of cataloging genetic variation from the Indian population, which have suggested the presence of distinct genome-level substructuring and its probable impact on disease biology (The Indian Genome Variation Consortium, 2005; Narang et al., 2010; The HUGO Pan-Asian SNP Consortium, 2011; Upadhyay et al., 2016; Rustagi et al., 2017). However, there are a few limitations to these studies as they predominantly catalog germline variants designed to capture common high-frequency variations, which is sufficient for deciphering population structure, but lacks information on rare mutations and copy number variations (CNVs). Equally important, these are not available as an open-access reference map.

In this study, we report the development and completion of phase I of a new database—the INDian EXome database (INDEX-db), which catalogs genetic variations in exonic and regulatory regions from healthy control individuals across different geographical regions of southern India. The database is a comprehensive collection of different types of genetic variations viz. single-nucleotide polymorphisms (SNPs), small insertions and deletions, and CNVs identified from WES. The database is hosted online with a user-friendly interface to access, download, and query the information. The genetic variation data can be browsed using the genome browser integrated with the database. We believe that such an integrated reference database for this population may be valuable to understand the genomic architecture underlying susceptibility to disease, detect familial or geographical clustering of the population, and thus aid efforts to understand disease genetics.

## 2. Methods

### 2.1. Sample information and ethical approval

Thirty-one individuals considered asymptomatic for any common adult-onset clinical illness (as per interviews and records) were selected for the study at the National Institute of Mental Health and Neuro Sciences, Bengaluru, India. The study was approved by the institutional ethics committee. Written informed consent was obtained from all participants before sampling. Ten microliters of peripheral blood was collected under aseptic conditions and high-molecular-weight DNA was isolated.

### 2.2. Library preparation and exome sequencing

The genomic DNA was extracted from blood and the Illumina Nextera Rapid Capture Expanded Exome kit was used for library preparation. Sequencing was carried out on the Illumina HiSeq NGS platform. Quality check of raw reads was performed using the FASTQC tool (www.bioinformatics.babraham.ac.uk/projects/fastqc/). Only paired-end raw reads with a score more than Q20 were filtered using Prinseq lite, version 0.20.4 (Schmieder and Edwards, 2011), for further alignment to the reference genome. Reads were also checked for per-base and per-sequence quality scores, GC (Guanine-Cytosine) content, and sequence length distribution.

### 2.3. Alignment and mapping of reads

The raw reads were aligned to the human reference genome hg19 (GRCh37) using the BWA tool, version 0.5.9 (Li and Durbin, 2009). Polymerase chain reaction duplicates in the mapped reads were marked using Picard (http://broadinstitute.github.io/picard/). Indel realignment was performed using GATK, version 3.6 (Depristo et al., 2011). Conversion of the sequence alignment file [Sequence Alignment Map (SAM) to Binary Alignment Map (BAM)], indexing, and sorting were done by SAMtools, version 1.5 (Li et al., 2009). The quality check for alignment on mapped reads was performed using Qualimap, version 2.2.1 (Okonechnikov et al., 2015).

### 2.4. Detecting single-nucleotide polymorphisms, indels, and copy number variations

SNPs and short insertions/deletions (indels) were called from the aligned files using Varscan2, version 2.3.9 (Koboldt et al., 2009, 2012) (with the criteria of min coverage = 8, Minor Allele Frequency (MAF) ≥0.25%, and *p*-value ≤0.001). The depth of coverage was calculated using GATK, version 3.8.0 (16), and this was used to detect CNVs using XHMM (Fromer et al., 2012; Fromer and Purcell, 2014). XHMM employs principal component analysis to remove batch and target effects. Principal component analysis was performed on the entire read–depth matrix (31 individuals by 336,037 targets), and a hidden Markov model was applied to the normalized data to detect CNVs. Functional impact of genetic variants was analyzed using two in silico algorithms, SIFT (Ng and Henikoff, 2003) and PolyPhen2 (Adzhubei et al., 2010), which predict the functional consequences of variations.

### 2.5. Haplotype phasing

Haplotype prephasing was done for SNP genotypes from 31 individuals using SHAPEIT2 (v2.r837.GLIBCv2.12) (Delaneau et al., 2014; O'Connell et al., 2014). As a haplotype reference, we downloaded the 1000 Genomes project Phase3 reference (http://mathgen.stats.ox.ac.uk/impute/1000GP_Phase3/) and used only the SAS subgroup haplotype reference. The phased data were visualized and haplotype blocks were generated based on Dprime values computed for every comparison between markers (SNPs), which are present within a distance range of 500 kb using Haploview, version 4.2 (Barrett et al., 2005). Default parameters were used, which include markers having MAF values >0.05, *p*-value cutoff of 0.001, with maximum Mendelian errors of 1, minimum genotype percentage of 75%, and exclusion of individuals with >50% of missing genotypes, with 95% confidence bounds (Gabriel et al., 2002).

### 2.6. Development of the INDian EXome database

**FIG. 1.**
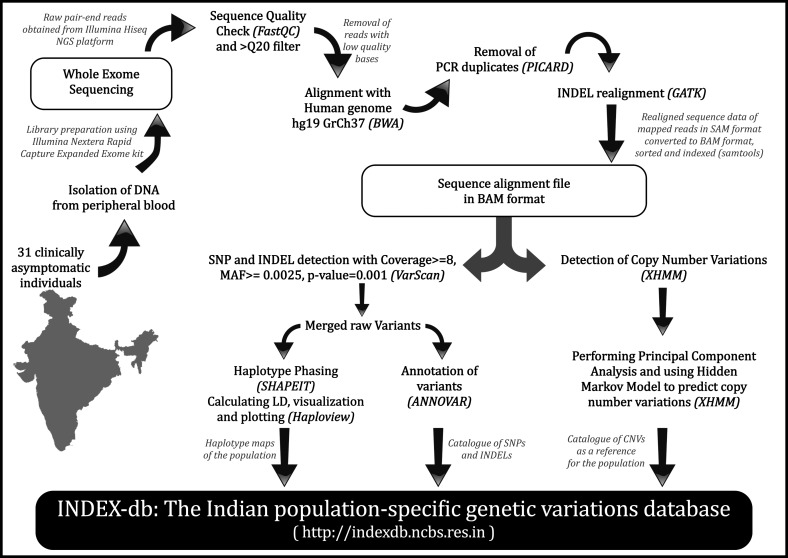
The workflow of development of phase I of INDEX-db. The steps involved in development of INDEX-db. The tools used in every step are mentioned in brackets.

The SNPs and indels obtained from all 31 individuals were merged using VCFtools (Danecek et al., 2011) to create a merged SNP and indel catalog. This was annotated with ANNOVAR (reference assembly 65) (Wang et al., 2010). CNVs were pooled from all the individuals and used to create a reference copy number profile for the population. Pooling of data, functional analysis, and other downstream analysis were performed using in-house shell and Python scripts. The entire workflow of developing INDEX-db is shown in Figure 1.

### 2.7. Database architecture and webpage implementation

The INDEX-db was developed using the LAMP (Linux, Apache, MySQL, and PHP/Python) architecture. The webpage of the INDEX-db was implemented using HTML5 and CSS3. Query tools were implemented by using Python as the common gateway interface. The graphical genome browser for the database was developed on JBrowse, version 1.12.3 (Skinner et al., 2009).

### 2.8. Data availability

The raw sequence data have been deposited at the NCBI SRA database (SRA accession SRP135959). The entire database is hosted online at http://indexdb.ncbs.res.in and is freely accessible along with associated tools for querying and comparing user data with INDEX-db. The data are also available for download in standard formats at http://indexdb.ncbs.res.in/downloads.html The SNPs are also deposited at the NCBI's dbSNP (https://www.ncbi.nlm.nih.gov/SNP/snp_viewTable.cgi?handle=OMUKHERJEE_ADBS).

## 3. Results

### 3.1. Database content and features

**FIG. 2.**
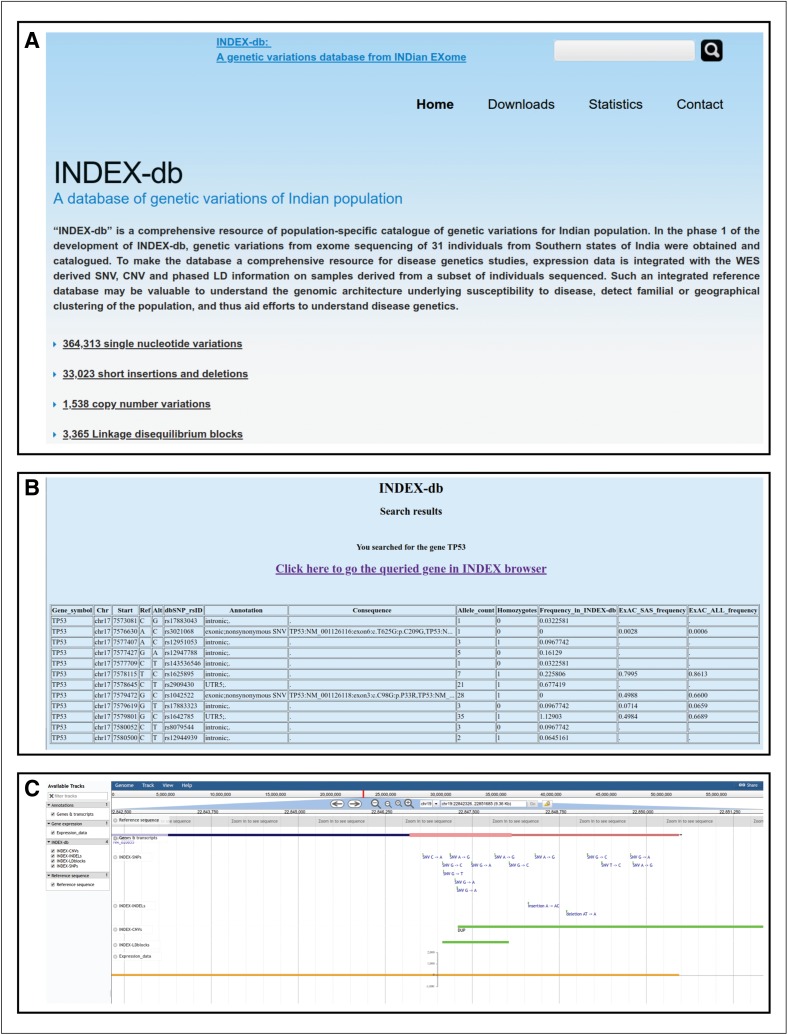
INDEX-db web implementation. **(A)** A screenshot of the INDEX-db home page. **(B)** A screenshot of results of a gene query in the database. The result includes the list of variants in the gene, their frequency in the INDEX database, and other associated information. The link to visualize the gene and genetic variations in the genome browser is generated and provided in the query result page. **(C)** A screenshot of the INDEX genome browser, with tracks of SNPs, indels, CNVs, and LD blocks. CNV, copy number variation; LD, linkage disequilibrium; SNP, single-nucleotide polymorphism.

The database provides download, query, and genome browser modules. The modules are placed in a user-friendly and responsive web interface of the database (Fig. 2A). The user will be able to download the entire processed dataset in standard formats. The querying module allows the user to identify the list of all genetic variants in a given gene. It also allows querying for a specific variant using the dbSNP identifier (rsID). The query results provide the frequency of the queried variants along with other annotations (Fig. 2B). The genome browser allows the user to visualize the genetic variation data. The browser consists of tracks for SNPs, indels, CNVs, and haplotype blocks, along with annotation track of genes and transcripts (Fig. 2C). The browser includes few more features such as highlight regions of the genome and share by generating a link, upload new track files to compare, and provide complete annotation information of each variant when the user clicks on it.

### 3.2. INDEX-db: variation profile

**FIG. 3.**
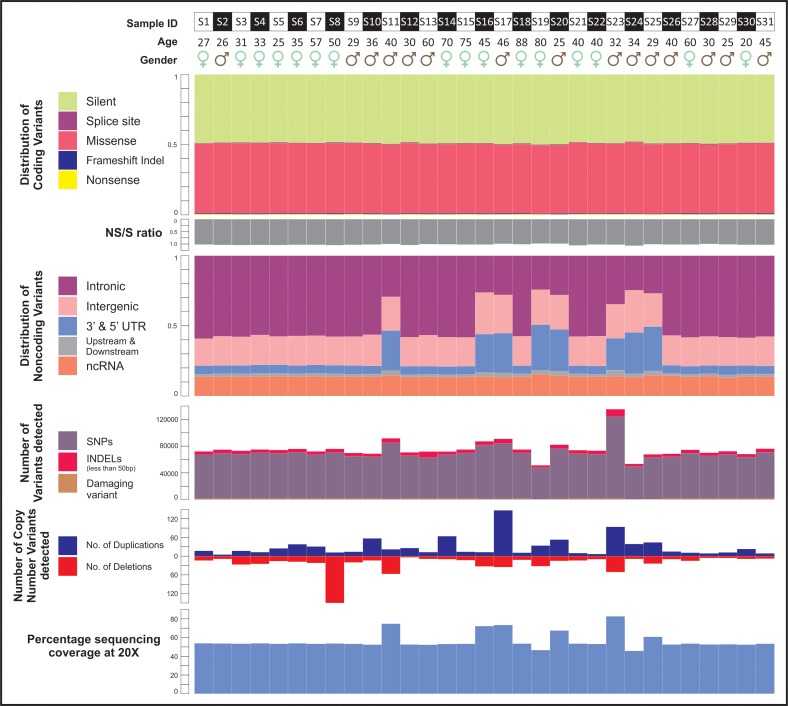
Variant profile. The distribution of coding and noncoding variants, the nonsynonymous-to-synonymous ratio, and the percentage coverage of sequencing at 20 × of 31 individuals cataloged in INDEX-db. The number of SNPs and CNVs detected in every individual. ncRNA, noncoding RNA; NS, nonsynonymous; S, synonymous; UTR, untranslated region.

**FIG. 4.**
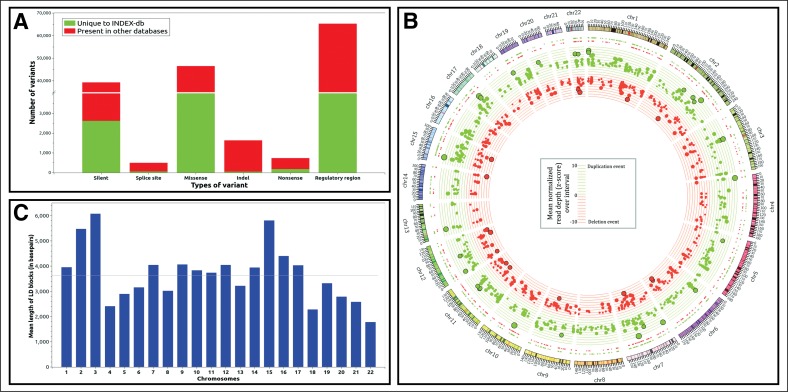
INDEX-db genetic catalog. **(A)** Comparison of INDEX-db with other public databases. **(B)** The circos plot showing the copy number variation events cataloged in INDEX-db. Duplication and deletion events have been colored green and red, respectively. **(C)** Mean length of linkage disequilibrium blocks identified in autosomes.

A total of 397,336 single-nucleotide variations and short insertions/deletions were identified in this phase I of INDEX-db, with an average 96% of the reads mapping to the reference genome at a mean coverage of 54.6% with at least 20 × depths (Fig. 3). There was no significant bias seen in terms of sequencing and/or sample QC (Quality Control) (Fig. 3). About ∼23% of the total genetic variations identified were in the coding region, of which nearly half (51.34%) were missense variations, followed by silent (43.36%), indel (1.8%), nonsense (0.81%), and splice sites (0.55%) (Fig. 4A). The ratio of nonsynonymous (NS = 49013) to synonymous variants (*S* = 39,876) was 1.23 (Fig. 3). The SNP profile observed in our study is comparable with exome sequencing reports published earlier (Lek et al., 2016; Upadhyay et al., 2016; Rustagi et al., 2017).

CNVs contribute about one-tenth of a percent (0.1%) of the total genetic variations of an individual and they affect longer regions than both SNPs and short indels (The 1000 Genomes Project Consortium, 2015). CNVs have a spectrum of phenotypic effects, from adaptive traits (Beckmann et al., 2007) to embryonic lethality (Hurles et al., 2008), and are implicated in many disorders, including schizophrenia (Cook and Scherer, 2008), Down's syndrome (Korenberg et al., 1994), kidney diseases (Nagano et al., 2018), diabetes (Prabhanjan et al., 2016; Ascencio-Montiel et al., 2017), hypertension (Marques et al., 2014; Boon-Peng et al., 2016), cancer (Liu et al., 2013; Araujo et al., 2014), and bipolar disorder (Grozeva et al., 2013). Using a hidden Markov model-based tool, we identified a total of 1538 CNVs in the size range of 50 bp to 3 mb in the INDEX-db phase I analysis, represented as a circos plot (Fig. 4B). The number, size range, and distribution of detected CNVs in INDEX-db are comparable with other published data (MacDonald et al., 2014).

The common pattern in which variants are inherited across a population has critical importance in studying genetic correlates of rare and complex human diseases (The International HapMap Consortium, 2005). As parental genotype information may not be available for all samples, references of phased haplotypes imputed using population-relevant references are thus valuable for disease genetic investigations. We could identify a total of 3365 linkage disequilibrium (LD) blocks spread across the autosomes with an average block length of ∼3.6 kb (Fig. 4C).

### 3.3. Comparison of INDEX-db with other public databases

Population genetic studies have shown that there is a greater genetic drift in East Asian populations, and this has an impact on the number of variants detected in an individual (Balick et al., 2015; Gao and Keinan, 2016; Simons and Sella, 2016). To ascertain the value of INDEX-db as a reference resource for disease genetic studies for the Indian population, we compared the INDEX-db phase I data with two publicly available databases. We used the Exome Aggregation Consortium (ExAC) (Lek et al., 2016) as it is one of the largest exome sequencing reference databases with significant representation of the South Asian population (although low representation from the pan-Indian population) and the Andhra Pradesh-South Asian Samples (AP-SAS) (Rustagi et al., 2017) as it is a WGS- (Whole Genome Sequencing)-based dataset generated using samples from southern India. We found that 12% (48,732) of the variants identified were unique to INDEX-db phase I (Fig. 4A and Supplementary Table S1). Within the coding region, this translated to 8860 (∼2.23%) variations, of which 966 had a functional annotation of being deleterious by two in silico algorithms, SIFT and PolyPhen2 (Ng and Henikoff, 2003; Adzhubei et al., 2010). We found ∼20% of coding variants identified in INDEX-db were in common with ExAC and ∼7% with AP-SAS. The observation of low overlap between INDEX-db and AP-SAS could be attributed to the low coverage in the whole-genome sequencing design of the AP-SAS study (∼2 × mean coverage).

Differences between ExAC-SAS and INDEX-db could perhaps be attributed to the population-specific variation signature, especially since ExAC-SAS has a low representation from the Indian population. The mutational profile obtained in phase I of this database is comparable, overall, with other databases, although it is currently limited by the number of individuals it represents.

## 4. Discussion

We report the development of a new database, INDEX-db, which summarizes variations in coding and regulatory regions identified from healthy control individuals. The first phase of the database consists of data of 31 individuals from southern India. The database is layered with information regarding CNVs and phased LD mapping. The integrated database is available freely at http://indexdb.ncbs.res.in along with associated tools for querying and comparing user input data with INDEX-db.

The INDEX-db is in its first phase, in comparison with other public databases, limited in terms of the number of individuals sequenced to represent the population. However, the variant profile we report in our pilot phase is comparable with population-based databases, signifying its value in terms of giving population-specific information.

The genetic basis of complex disorders needs to be better understood in India, where the number of individuals affected by these disorders is expected to increase significantly in the coming decades. In this context, we suggest that an integrated reference database may be valuable to understand the genomic architecture underlying susceptibility to disease and familial or geographical clustering of the population and thus aid our understanding of the disease.

## Supplementary Material

Supplemental data
