# Supplementary material for: INDEX-db: The Indian Exome Reference Database (Phase I)
Source: J Comput Biol. 2019 Mar 6;26(3):225–34. doi: 10.1089/cmb.2018.0199 (PMC6441288; doi:10.1089/cmb.2018.0199)
Supplement: Supplemental data [file Supp_Table1.pdf]

Supplementary Material

Supplementary Table S1. Comparison of INDEX-db with Other Public Databases

	Full INDEX-db	%	*ExAC-SAS and INDEX-db—Common*	%	*ExAC-SAS and INDEX-db—Unique*	%	*ExAC-full and INDEX-db—Common*	%	*ExAC-full and INDEX-db—Unique*	%	*SAS-AP and INDEX-db—Common*	%	*SAS-AP and INDEX-db—Unique*	%	*Total Unique in INDEX-db*	%
Coding																
Silent	39,316	9.89	34,900	8.78	4416	1.11	36,687	9.23	2629	0.66	13,355	3.36	25,961	6.53	2629	0.66
Splice site	499	0.13	303	0.08	197	0.05	434	0.11	66	0.02	61	0.02	439	0.11	66	0.02
Missense	46,550	11.72	37,360	9.4	9190	2.31	40,826	10.27	5724	1.44	11,133	2.8	35,417	8.91	5724	1.44
Indel	1637	0.41	465	0.12	1172	0.29	1581	0.4	56	0.01	74	0.02	1563	0.39	56	0.01
Nonsense	735	0.18	454	0.11	281	0.07	547	0.14	188	0.05	91	0.02	644	0.16	188	0.05
Unknown	1929	0.49	1500	0.38	429	0.11	1732	0.44	197	0.05	394	0.1	1535	0.39	197	0.05
Total coding	90,666	22.82	74,982	18.87	15,685	3.95	81,807	20.59	8860	2.23	25,108	6.32	65,559	16.5	8860	2.23
Noncoding																
Intron	134,278	33.79	54,980	13.84	79,298	19.96	126,154	31.75	8124	2.04	52,848	13.3	81,430	20.49	8124	2.04
Intergenic	67,814	17.07	4647	1.17	63,168	15.9	50,930	12.82	16,885	4.25	12,625	3.18	55,190	13.89	16,885	4.25
3′ UTR	43,816	11.03	4365	1.1	39,451	9.93	41,842	10.53	1974	0.5	19,704	4.96	24,112	6.07	1974	0.5
5′ UTR	10,605	2.67	2967	0.75	7638	1.92	10,041	2.53	564	0.14	3771	0.95	6834	1.72	564	0.14
Upstream	6462	1.63	666	0.17	5796	1.46	4984	1.25	1478	0.37	1640	0.41	4822	1.21	1478	0.37
Downstream	4417	1.11	401	0.1	4016	1.01	3410	0.86	1007	0.25	1303	0.33	3114	0.78	1007	0.25
ncRNA	39,278	9.89	6825	1.72	32,453	8.17	29,438	7.41	9840	2.48	9327	2.35	29,951	7.54	9840	2.48
Total noncoding	306,670	77.18	74,851	18.84	231,820	58.34	266,799	67.15	39,872	10.03	101,218	25.47	205,453	51.71	39,872	10.03
Total SNV	397,336	100	149,833	37.71	247,505	62.29	348,606	87.74	48,732	12.26	126,326	31.79	271,012	68.21	48,732	12.26

The "%" columns show the percentage with respect to the total number of variants in INDEX-db (397,336).
